# *Cinnamomum cassia* and *Rosa laevigata* Mixture Improves Benign Prostatic Hyperplasia in Rats by Regulating Androgen Receptor Signaling and Apoptosis

**DOI:** 10.3390/nu15040818

**Published:** 2023-02-05

**Authors:** Myunghee Kim, Phuong Tran, Jun Yin, Jungbin Song, Hocheol Kim

**Affiliations:** 1 NEUMED R&BD Institute, NeuMed Inc., 88 Imun-ro, Dongdaemun-gu, Seoul 02440, Republic of Korea; 2 Department of Herbal Pharmacology, College of Korean Medicine, Kyung Hee University, 26 Kyungheedae-ro, Dongdaemun-gu, Seoul 02447, Republic of Korea

**Keywords:** *Cinnamomum cassia*, *Rosa laevigata*, benign prostate hyperplasia, apoptosis, androgen receptor

## Abstract

Benign prostatic hyperplasia (BPH) is the most common condition in elderly men that is characterized by an increase in the size of the prostate gland. *Cinnamomum cassia* and *Rosa laevigata* have been reported to treat the symptoms associated with BPH. The aim of this study was to evaluate the effects of HT080, an herbal extract of *C. cassia* and *R. laevigata*, on a testosterone propionate (TP)-induced BPH rat model. The rats received a daily subcutaneous injection of TP (3 mg/kg) for 4 weeks to induce BPH. Rats were divided into four groups: group 1 (sham), group 2 (BPH, TP alone), group 3 (Fina, TP + finasteride 1 mg/kg/day), and group 4 (HT080, TP + HT080 200 mg/kg/day). At the end of the experiment, all rats were sacrificed, and their prostate glands were removed, weighed, and subjected to histopathological examination and western blot analyses. Serum testosterone and dihydrotestosterone (DHT) levels were determined. In addition, serum alanine and aspartate aminotransferase levels were measured to evaluate the toxicity in the liver. The Hershberger bioassay was also conducted to investigate the effects of HT080 on androgenic and antiandrogenic activities. In the BPH model, the prostate weight, prostate index, prostate epithelial thickness, and serum testosterone and DHT levels in the HT080 group were significantly reduced compared to the BPH group. Histological studies showed that HT080 reduced prostatic hyperplasia. The protein expression of androgen receptor from the HT080 group was significantly reduced in comparison with the BPH group (*p* < 0.05). HT080 also induced apoptosis by regulating Bcl-2 and Bax expression. In addition, HT080 showed no toxicity in the liver and did not exhibit androgenic and antiandrogenic activities. Our finding revealed that HT080 can be a potential candidate for the treatment of BPH by regulating androgen receptor signaling and apoptosis.

## 1. Introduction

Benign prostate hyperplasia (BPH) or prostate gland enlargement is the most common condition in middle-aged and elderly men regardless of their culture or ethnic origins [1,2]. It is characterized by an increase in the size of the prostate gland and not cancer-causing lower urinary tract symptoms (LUTSs) such as nocturia, weak urinary stream, urgency, and hesitancy [3,4]. It has been reported that the risk of BPH increases with age from 8% at age 31 to 40 years to 40–50% at age 51 to 60 years and to over 80% at age 80 years [5,6]. Much research revealed that risk factors for BPH and LUTS include hormonal alterations, obesity, diet-induced hyperinsulinemia, inflammation, lack of exercise, or glucose homeostasis as hyperglycemia [7,8,9,10,11,12]. Among them, hormonal alterations are considered the main causes of BPH, which leads to the imbalance of growth and apoptosis of prostate cells. Testosterone and dihydrotestosterone (DHT) are two main androgens that play a crucial role in the development of BPH [13]. DHT is an active metabolite of testosterone and is the most potent androgen in men. DHT converted from testosterone by 5-α-reductase binds to the androgen receptor (AR) with high affinity [14]. The binding of DHT to AR induces the proliferation of prostate cells via growth factors [15,16]. Over the past several years, the incidence of BPH increased rapidly as the population ages, which diminished health-related quality of life and increased the burden of medical expenses [17].

There are several treatments for BPH such as medications, minimally invasive therapies, and surgery. Surgical treatment by the transurethral resection of the prostate was used as the gold standard for the management of BPH. However, surgery can cause complications, including temporary difficulty when urinating, urinary tract infection, dry orgasm, impotence, heavy bleeding, urinary incontinence, low sodium in the blood, and the need for re-treatment. For this reason, medical therapy is preferred and safer in older adults. Currently, 5α-reductase inhibitors and α_1A_-adrenergic receptor blockers are the two main drug groups used for the treatment of BPH [18,19]. Finasteride and dutasteride, two primary 5α-reductase inhibitors, have been reported with various side effects during long-term administration such as sexual adverse effects, erectile dysfunction, and loss of libido [20,21,22]; these drugs decreased ejaculation by 3.4 to 15.8 percent in men [23]. In addition, the use of 5α-reductase inhibitors for BPH can be the reason for prostate cancer and the risk of all-cause mortality [24,25]. Moreover, tamsulosin, an α_1A_-adrenergic receptor blocker, may increase the risk of dementia in older men with BPH via its selectivity relative to α_1A_-adrenoceptors in the brain [26,27]. Therefore, the development of natural products from herbal plants for the treatment of BPH has received substantial attention due to being safer and less toxic than synthetic materials [28,29,30].

*Cinnamomum cassia* and *Rosa laevigata* are two herbs traditionally used to treat LUTS in Korea and China [31,32]. *C. cassia* originated from Southern China and is now widely grown in Eastern and Southern Asia; bark and stem are the two main used parts. Among several components of *C. cassia* such as terpenoids, phenylpropanoids, glycosides, and lignan, phenylpropanoids (cinnamaldehyde, cinnamic acid) are the major bioactive components of *C. cassia* bark [33,34,35]. *C. cassia* exhibited various pharmacological effects including anti-tumor, anti-inflammatory, anti-diabetic, antibacterial, antiviral, cardiovascular protective, cytoprotective, and neuroprotective effects [36]. *R. laevigata* is a climbing plant that originated from South China and Southeast Asia. The main fruit component includes triterpenoids, tannins, flavonoids, lignans, and polysaccharides [37]. The extracts and purified compounds of *R. laevigata* demonstrated various pharmacological effects including antioxidant activity, immunomodulatory, anti-inflammatory, liver protection, kidney protection, cardiovascular protection, and neuroprotective effects [37]. It has been reported that *R. laevigata* water extracts can decrease the frequency, prolong the interval of urination, and increase the quantity of each urination on the mouse pollakiuria model by severing the hypogastric nerve [38]. 

A previous study reported that cinnamic acid showed the antagonism of α_1A_-adrenoceptors [39], which have a role in prostatic smooth muscle tone regulation and are important mediators in LUTS and BPH. In addition, ellagic acid, the main component of *R. laevigata* fruits, has been reported for improving BPH [32]. Based on these previous studies and the traditional use of *C. cassia* bark and *R. laevigata* fruit, we assumed that the combined extracts of these two herbs (HT080) may show potential in the treatment of LUTS and BPH. To date, no scientific study assessing the efficacy and mechanism of HT080 in the treatment of BPH has been reported in animal experiments.

The objective of this study was to evaluate the effects of HT080 against BPH development and its underlying mechanisms using testosterone propionate (TP)-induced BPH rats. In addition, the effects of HT080 on the activities of androgenic and antiandrogenic were evaluated by the Hershberger bioassay.

## 2. Materials and Methods

### 2.1. Preparation of HT080

Dried *C. cassia* bark and *R. laevigata* fruits were purchased from Kyung Dong market (Buyoung, Daewoo, Seoul, Korea). These two herbs were identified by Prof. Dr. Hocheol Kim, and voucher specimens were placed at the Department of Herbal Pharmacology, College of Korean Medicine, Kyung Hee University (Seoul, Korea). Dried *C. cassia* was extracted twice in distilled water at 100 °C for 2 h (first extract) and then for 1 h (second extract). The solution was filtered and concentrated under reduced pressure and freeze-dried to obtain the powder extract. The dried *R. laevigata* was extracted in distilled water at 100 °C for 6 h. The solution was filtered and concentrated under reduced pressure and spray-dried with 60% dextrin. The dried extract powders of *C. cassia* and *R. laevigata* were mixed at a ratio of 17:83 (*w*/*w*), which is HT080.

### 2.2. High-Performance Liquid Chromatography (HPLC) of HT080 Extract

The quality of HT080 was controlled by measuring the contents of cinnamic acid and ellagic acid, which are marker compounds of *C. cassia* and *R. laevigata*, respectively. *R. laevigata* contains ellagitannins such as *laevigatins* E, F, and G that can be hydrolyzed into ellagic acids, which were determined as the total ellagic acid using HPLC [40]. Reference standards of cinnamic acid and ellagic acid were purchased from Nature Standard (Shanghai, China). For analyzing the content of cinnamic acid in HT080, 0.1 g of HT080 was mixed with 10 mL of 50% methanol (*v*/*v*) and sonicated for 10 min followed by filtering through a 0.45 µm syringe filter before injecting into an HPLC system. For analyzing the content of total ellagic acid in HT080, HT080 (1 g) was hydrolyzed by 10 mL of refluxed (ethanol: distilled water: hydrochloride = 60:20:20) at 90 °C for 1 h and then allowed to cool to room temperature. The hydrolyzed solution was transferred to a 100 mL mass flask, and the reaction flask was washed with a small volume of methanol and combined with the previous reactant. After repeating this procedure 2 times and filled with 100 mL of methanol, the prepared solution was filtered using a 0.45 µm syringe filter before injecting into the HPLC system. The HPLC system (Agilent 1260, Agilent Technologies, Santa Clara, CA, USA) consisted of a G7111A quaternary pump, a G7155A diode array detector, and a G7129A autosampler. Mobile phase A consisted of 0.1% phosphoric acid in DW (*v*/*v*), and the mobile phase B was acetonitrile; an Eclipse Plus C_18_ column (4.6 × 250 mm i.d., 5 µm) was maintained at 35˚C. Gradient flows were as follows: 0–15 min, 18% B; 15–18 min, 18–38% B; 18–26 min, 38–40% B; 26–27 min, 40–100% B; 27–30 min, 100% B; 30–31 min, 100–18% B; 31–36 min, 18% B. The flow rate of 1.0 mL/min and an injection volume of 10 µL were set. The detector was set at 275 nm for cinnamic acid and 253 nm for ellagic acid. Each experiment was performed in triplicate. 

### 2.3. Animal Experimental Design

A total of 25 male Sprague–Dawley (SD) rats (ten weeks old) were purchased from Daehan BioLink (Eumseong, Chungbuk, Korea). Rats were housed in polycarbonate cages under controlled temperatures at 23 ± 1 °C, a humidity of 55–60%, and a 12/12 h light/dark cycle. Animals were fed standard chow (Samtako, Osan, Gyeonggi-do, Korea) and water *ad libitum*. After 1 week of adaptation, 19 rats except the sham group were castrated under isoflurane anesthesia (Forane, JW pharmaceutical, Seoul, Korea). The castration was conducted by excised testicles and epididymal fat from the abdomen according to a previous study [41]. After 1 week of castration, rats were randomly divided into 4 groups: In group 1 (sham, *n* = 6), rats were administered distilled water orally and olive oil subcutaneously (s.c); in group 2 (BPH, *n* = 6), rats were administered distilled water orally and 3 mg/mL/kg body weight (BW) of TP in olive oil (s.c); in group 3 (Fina, *n* = 6), rats were administered 1 mg/10 mL/kg finasteride in distilled water (Proscar^®^, MSD, Seoul, Korea) orally and 3 mg/mL/kg of TP in olive oil (s.c); in group 4 (HT080, *n* = 7), rats were administered 200 mg/10 mL/kg of HT080 in distilled water orally and 3 mg/mL/kg of TP in olive oil (s.c). The treatment was conducted for 28 days, and all treatment volumes were adjusted after weighing each rat daily. After 4 weeks, the animals were fasted overnight and sacrificed under isoflurane anesthesia. Blood samples were taken from the vena cava, left at room temperature for 60 min, and centrifuged at 4000 rpm at 4 °C for 10 min. The separated serum was stored until a further study at −80 °C. After animals were sacrificed, prostate tissues were instantly excised and weighed. The prostate index (PI) was determined as the ratio of prostate weight (mg) to 100 g of BW. One side of the prostate ventral lobe sections was fixed with 4% paraformaldehyde for histological analysis; the other side of the prostate lobe was stored at −80 °C and used for western blotting analyses. The protocol was performed according to the animal care guidelines of the institutional animal care and the use committee of the NEUMED R&DB Institute (protocol no. KISTEM-IACUC-2021-001).

### 2.4. Histology

Prostate tissue was embedded in paraffin and cut to thick sections of 4 μm and then stained with Harris’ hematoxylin-eosin. Subsequently, the sections were mounted and cover slipped by the mounting solution. A histological examination of prostatic sections was evaluated by a microscope (Nikon, Tokyo, Japan). The epithelial thickness (in 10 random sites) was determined by calculating the basal pole and the apical pole’s distance (×200). The average epithelial thickness value in 10 random sites was used. 

### 2.5. Determination of Testosterone and DHT Levels in Serum

Serum concentrations of testosterone and DHT were measured using enzyme-linked immunosorbent assay kits (Cusabio, Houston, TX, USA). The assay was conducted according to the instruction of the manufacturer. 

### 2.6. Measurement of Serum Alanine Aminotransferase (ALT) and Aspartate Aminotransferase (AST) Levels

Serum ALT and AST levels were measured to assess the liver toxicity using the FUJI DRI-CHEM SLIDE kit and the FUJI DRI-CHEM NX700 analyzer (Fujifilm, Tokyo, Japan).

### 2.7. Western Blot Analysis

Western blotting was performed to assess BPH-related protein expression. The prostatic tissue samples were homogenized in a RIPA buffer (Thermo Scientific, Waltham, MA, USA) with Bead Ruptor 24 Elite (OMNI International. Kennesaw, GA, USA) and centrifuged at 13,000 rpm for 3 min at 4 °C. The supernatant was collected in a clean tube to determine the protein concentration using the Pierce™ BCA Protein Assay Kit (Thermo Scientific, Waltham, MA, USA). Protein in each sample was quantified to 60 μg and heated for 5 min at 100 °C before being separated on Bolt™ 4–12% Bis-Tris Plus gels (Invitrogen, Waltham, MA, USA). After gel electrophoresis (100 V, 2 h), proteins were transferred to PVDF membranes (20 V, 2 h) and then blocked in 5% skim milk for 1 h. Subsequently, the membranes were incubated overnight at 4 °C with primary antibodies against AR (1:200, Santa Cruz Biotechnology, Dallas, TX, USA), Bcl-2 (1:1000, Abbkine, Wuhan, China), Bax (1:1000, Abbkine, Wuhan, China), and β-actin (1:500, Santa Cruz Biotechnology, Dallas, TX, USA). The primary antibody was removed by washing three times with TBST containing 1% Tween-20 (Biosolution, Suwon, Korea) and incubated with the secondary antibody, Goat anti-Mouse lgG (H+L) (1:5000 for β-actin and Bcl-2, 1:2000 for AR and Bax), at room temperature for 1 h. The membranes were washed 3 times and developed using the WesternBright ECL HRP substrate (Advansta, San Jose, CA, USA). The chemiluminescence of the membranes was imaged with an Amersham Imager 600 (GE Healthcare Life Sciences, Chicago, IL, USA), and protein expression was analyzed in Image J software (ver. 1.52, National Institutes of Health, Bethesda, MD, USA) and expressed relative to the total protein content in the sample.

### 2.8. Hershberger Bioassay

The Hershberger bioassay was conducted according to OECD guidelines (2009). SD rats (n = 30, 5 weeks old) were obtained from Daehan BioLink (Eumseong, Chungbuk, Korea). Animals were acclimated in polycarbonate cages under controlled temperatures at 23 ± 1 °C, a humidity of 55–60%, and a 12/12h light/darkness cycle. Animals were fed with a commercial standard chow (Samtako, Osan, Gyeonggi-do, Korea) and water *ad libitum*. After 1 week of adaptation, animals were castrated and allowed to recover for a week. Then, rats were randomly divided into six groups (control (n = 6), positive control (n = 6), HT080 100 mg/kg (n = 6), HT080 300 mg/kg (n = 6), HT080 1000 mg/kg (n = 6), and HT080 3000 mg/kg (n = 6)) that received respective treatments daily for 10 consecutive days. The control group was administered distilled water orally and olive oil s.c. For the other 5 groups, TP was administered daily by s.c injections at a dose of 0.4 mg/kg/day in olive oil (1 mL/kg). Rats in the positive control received flutamide (Sigma, St. Louis, MO, USA) orally at a dose of 3 mg /kg/day in a volume of 10 mL/kg. Other test substance groups received HT080 orally at a dose of 100, 300, 1000, and 3000 mg/kg/day in a volume of 10 mL/kg. After the final administration, rats fasted overnight before sacrifice. Five androgen-dependent tissues including ventral prostate (VP), seminal vesicles (SV), levator ani-bulbocavernosus muscle (LABC), Cowper’s glands (COW), and glans penis (GP) were harvested and weighed separately and compared to the control group. The relative organ weights were calculated based on the fasted body weight.

### 2.9. Statistical Analysis

Data were analyzed using a one-way analysis of variance (ANOVA) followed by Dunnett’s test in GraphPad Prism 7 software (GraphPad Software, Inc., San Diego, CA, USA). Statistical significance was set at *p* < 0.05 (^#^ *p* < 0.05, ^##^ *p* < 0.01, and ^###^ *p* < 0.001 vs. sham group; * *p* < 0.05, ** *p* < 0.01, and *** *p* < 0.001 vs. control group). All data are expressed as the mean ± standard deviation (S.D.).

## 3. Results

### 3.1. HPLC Analysis of Cinnamic Acid and Ellagic Acid in the HT080 Extract

**Figure 1:**
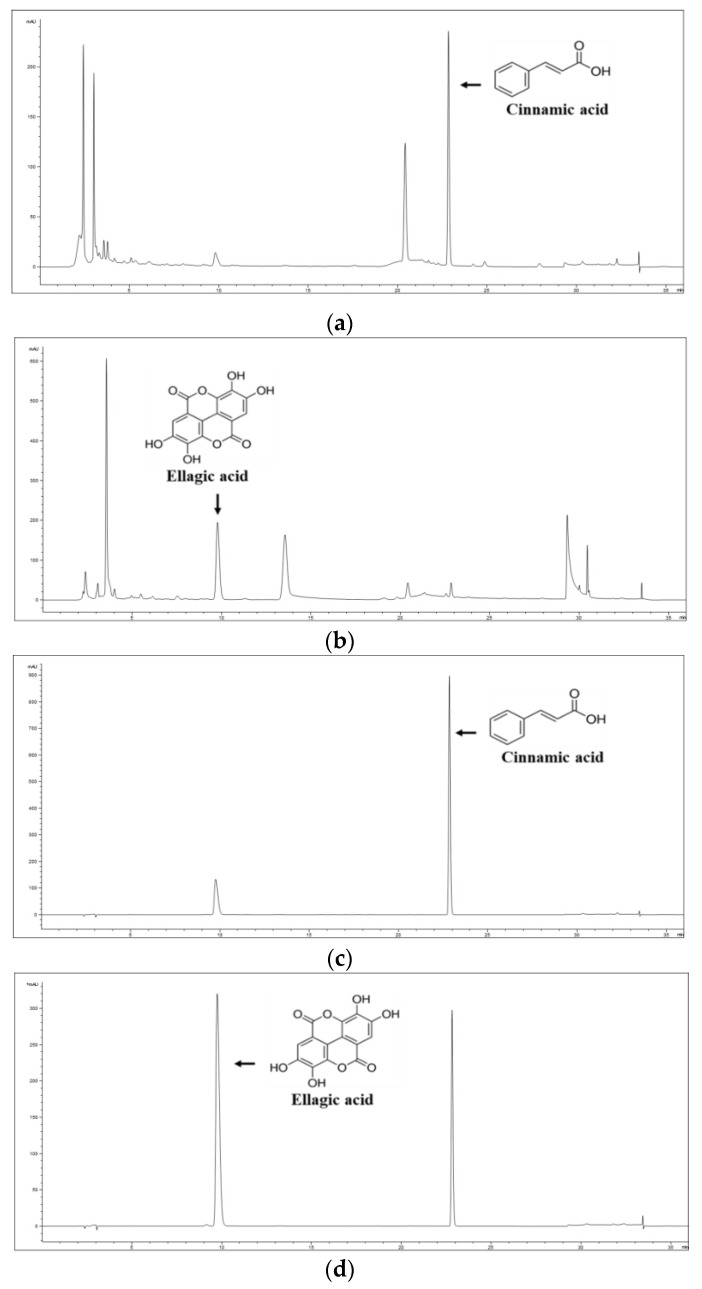
HPLC chromatogram of cinnamic acid (**a**) and ellagic acid (**b**) in the HT080 extract; reference standards for cinnamic acid (**c**) and ellagic acid (**d**).

The phytochemical compounds in the HT080 extract were identified using HPLC. The result is shown in Figure 1, with the two major active compounds being cinnamic acid and ellagic acid. The concentrations of cinnamic acid and ellagic acid in the HT080 extract were 1.43 ± 0.03 and 2.16 ± 0.01 mg/g, respectively. Cinnamic acid showed a characteristic peak at 22.64 min, and ellagic acid showed a characteristic peak at 9.37 min.

### 3.2. Effects of HT080 on Prostate Weight and PI

**Figure 2:**
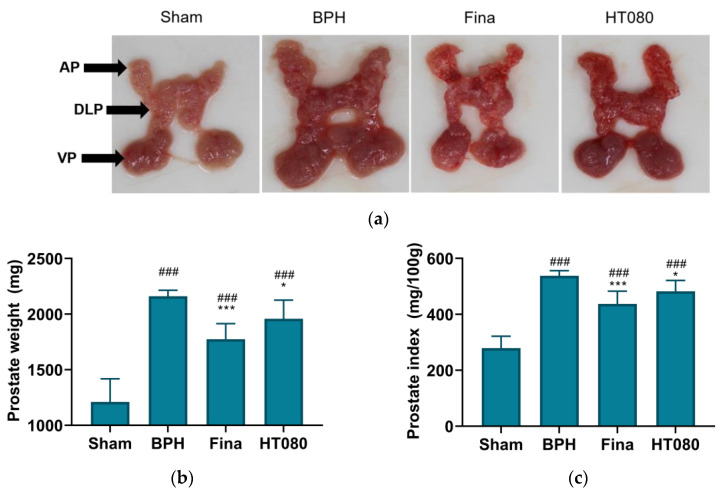
Effects of HT080 on prostate tissue in testosterone propionate-induced benign prostatic hyperplasia (BPH) rats. (**a**) Photographs of the prostate tissue (ventral prostate—VP; dorsolateral prostate—DLP; anterior prostate—AP). (**b**) Prostate weight of the rats. (**c**) Prostate index of the rats. Statistical significance: ^###^ *p* < 0.001 (compared to sham group using Dunnett’s test); * *p* < 0.05, *** *p* < 0.001 (compared to BPH group using Dunnett’s test). Values are expressed as the mean ± S.D. Fina, finasteride.

The effects of HT080 on prostate weight and PI are shown in Figure 2. Figure 2a shows the photographs of the prostate tissue, including ventral prostate (VP), dorsolateral prostate (DLP), and anterior prostate (AP); the prostate weight and PI of the rats are shown in Figure 2b,c, respectively. The volume and size of prostate tissues in the BPH group increased in comparison with the sham group, while the volume and size of prostate tissues in the finasteride and HT080 decreased compared to the BPH group (Figure 2a). The prostate weights of sham, BPH, finasteride, and HT080 groups were 1211 ± 206.9 mg, 2161 ± 52.8 mg, 1774 ± 140.4 mg, and 1958 ± 167.6 mg, respectively. The prostate weights of the BPH, finasteride, and HT080 groups significantly increased compared to the sham group (*p* < 0.001); the prostate weights of finasteride (*p* < 0.001) and HT080 (*p* < 0.05) groups were significantly reduced compared to the BPH group (Figure 2b). The PIs of sham, BPH, finasteride, and HT080 groups were 279.1 ± 42.6, 537.6 ± 18.8, 437.3 ± 45.3, and 482.0 ± 39.3 mg/100 g, respectively. The PI of the BPH group increased compared to the sham group (*p* < 0.001); the PIs of the finasteride (*p* < 0.001) and HT080 (*p* < 0.05) groups were significantly reduced in comparison with the BPH group (Figure 2c). 

### 3.3. Effects of HT080 on Prostate Tissue Histology

**Figure 3:**
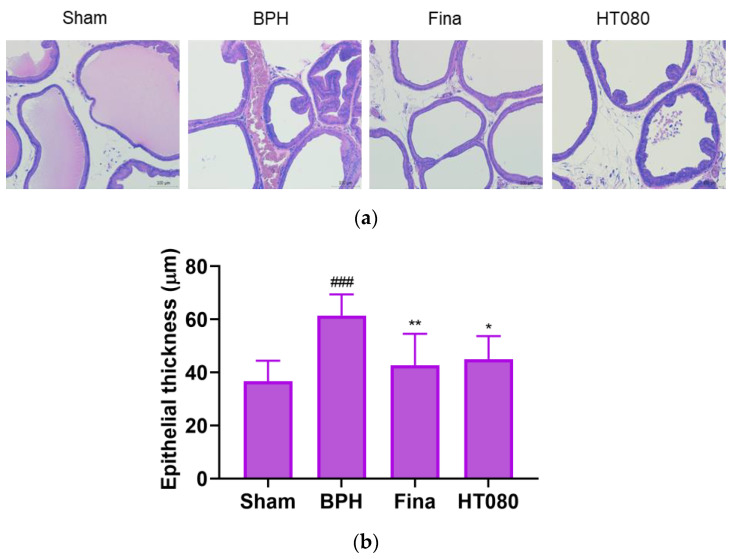
The histopathological analysis of the prostate tissues in testosterone propionate-induced benign prostatic hyperplasia (BPH) rats after being treated with HT080. (**a**) H&E staining of sham, BPH, finasteride (Fina), and HT080 groups. (**b**) Quantification of the thickness of epithelial layers. Statistical significance: ^###^ *p* < 0.001 (compared to sham group using Dunnett’s test); * *p* < 0.05, ** *p* < 0.01 (compared to BPH group using Dunnett’s test). Values are expressed as the mean ± S.D.

To confirm the effects of HT080 on alleviating prostatic hyperplasia, histological analysis was conducted using H&E staining. The morphological changes in prostate tissues are displayed in Figure 3. Morphological changes in the prostate tissue of each group are shown in Figure 3a. The typical features of prostatic hyperplasia related to the epithelial morphological change were clearly revealed in the BPH group. In comparison with the sham group, the prostatic tissue of the BPH group exhibited disturbances with respect to the shape and size of the prostatic tissue. Rats from the finasteride and HT080 groups exhibited a reduction in the size of the epithelial cell layer compared to the BHP group. The epithelial thickness is shown in Figure 3b. The prostatic epithelial thicknesses of sham, BPH, finasteride, and HT080 groups were 36.72 ± 7.74 μm, 61.41 ± 8.04 μm, 42.78 ± 11.76 μm, and 45.02 ± 8.68 μm, respectively. The prostatic epithelial thickness of the BPH group significantly increased in comparison with the sham group (*p* < 0.001). In contrast, prostatic epithelial cell layers from the finasteride and HT080 groups showed only mild epithelial hyperplasia which reduces the size and weight of the prostate in comparison with the BPH group. In addition, no significant difference in the prostatic epithelial thickness was observed when comparing the finasteride and HT080 groups with the sham group.

### 3.4. Serum Concentration of Testosterone and DHT

**Figure 4:**
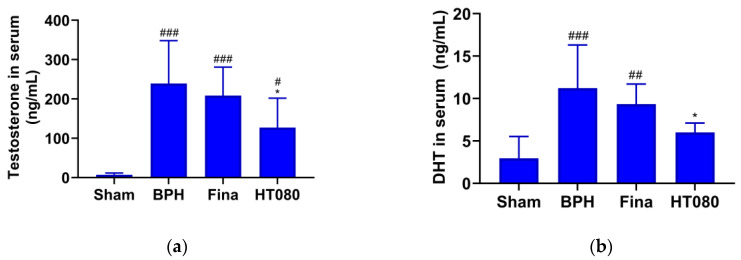
Concentration of testosterone (**a**) and DHT (**b**) in serum. Statistical significance: ^#^ *p* < 0.05, ^##^ *p* < 0.01, ^###^ *p* < 0.001 (compared to sham group using Dunnett’s test); * *p* < 0.05 (compared to BPH group using Dunnett’s test). Values are expressed as mean ± S.D. Fina, finasteride.

DHT is the main active metabolite of testosterone. The serum testosterone and DHT concentrations were determined and are shown in Figure 4. The serum testosterone concentrations were measured to be 7.06 ± 4.57 ng/mL, 239.2 ± 108.9 ng/mL, 208.4 ± 72.5 ng/mL, and 127.0 ± 74.9 ng/mL for the sham, BHP, finasteride, and HT080 groups, respectively (Figure 4a). The serum testosterone concentration of the BPH group significantly increased compared with the sham group (*p* < 0.001), while the serum testosterone concentration of the HT080 group significantly decreased in comparison with the BPH group (*p* < 0.05). The serum DHT concentrations were measured to be 2.96 ± 2.57 ng/mL, 11.2 ± 5.08 ng/mL, 9.34 ± 2.37 ng/mL, and 6.01 ± 1.11 ng/mL for the sham, BHP, finasteride, and HT080 groups, respectively (Figure 4b). The serum DHT concentration of the BPH group significantly increased compared with the sham group (*p* < 0.001), while the serum DHT concentration of HT080 group significantly decreased in comparison with the BPH group (*p* < 0.05).

### 3.5. Serum ALT and AST Levels

**Figure 5:**
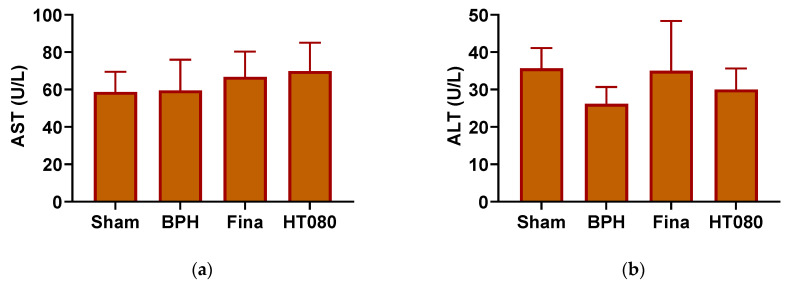
Levels of serum AST (**a**) and ALT (**b**) in sham, BPH, finasteride (Fina), and HT080 groups. Values are expressed as mean ± S.D.

The AST and ALT are serum enzyme markers of liver toxicity. The effects of HT080 on serum AST and ALT levels are shown in Figure 5. The AST values of the sham, BPH, finasteride, and HT080 groups were determined to be 58.7 ± 10.8 U/L, 59.5 ± 16.5 U/L, 66.8 ± 13.4 U/L, and 69.9 ± 15.2 U/L, respectively. The ALT values of the sham, BPH, finasteride, and HT080 groups were measured to be 35.7 ± 5.43 U/L, 26.2 ± 4.54 U/L, 35.0 ± 13.4 U/L, and 30.0 ± 5.63 U/L, respectively. No significant difference in AST and ALT levels was observed among the four groups, indicating normal liver function. 

### 3.6. Effects of HT080 on AR and Apoptosis in BPH-Induced Rats

**Figure 6:**
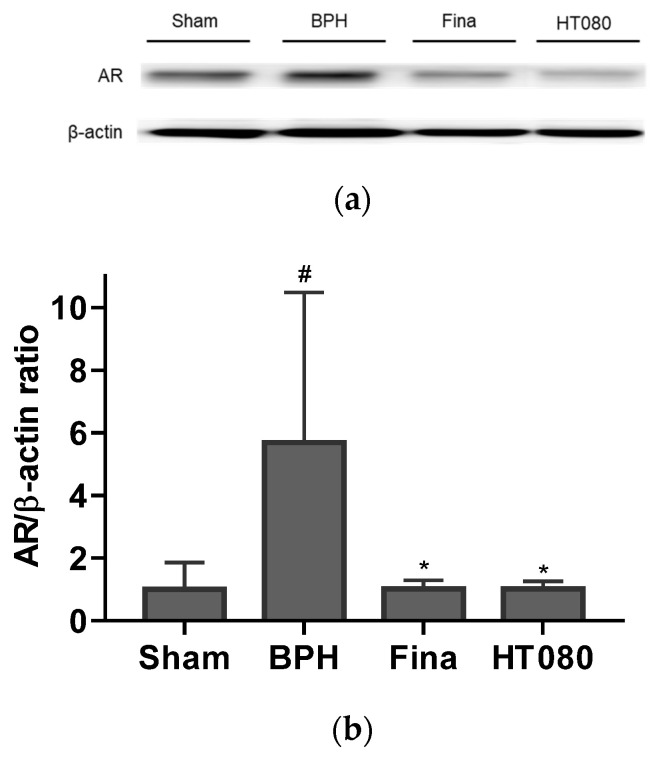
Effects of HT080 on the expression of androgen receptor (AR) via western blot: Representative western blot analysis of AR (**a**) and relative densitometry evolution of AR (**b**). Statistical significance: ^#^ *p* < 0.05 (compared to sham group using Dunnett’s test) and * *p* < 0.05 (compared to BPH group using Dunnett’s test). Values are expressed as the mean ± S.D. Fina, finasteride.

The effects of HT080 on the protein level of AR and apoptosis-related molecules were investigated in the prostate of BPH-induced rats. Figure 6 shows the effects of HT080 on AR expression. The AR expression from the BPH group significantly increased compared with the sham group (*p* < 0.05). As expected, the AR expression from the HT080 group was significantly reduced in comparison with the BPH group (*p* < 0.05). 

**Figure 7:**
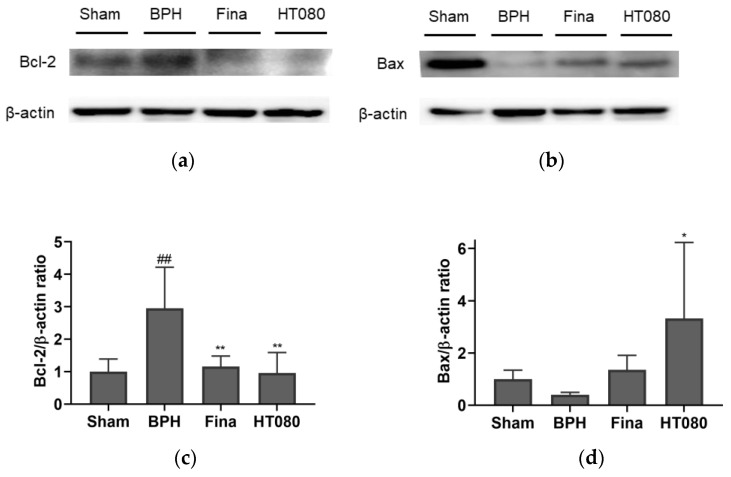
Effects of HT080 on the expression of Bax and Bcl-2 proteins via western blot: Representative western blot analysis of Bax (**a**) and Bcl-2 (**b**), relative densitometry evolution of Bcl-2 (**c**), and relative densitometry evolution of Bax (**d**). Statistical significance: ^##^ *p* < 0.01 (compared to sham group using Dunnett’s test); * *p* < 0.05 and ** *p* < 0.01 (compared to BPH group using Dunnett’s test). Values are expressed as the mean ± S.D. Fina, finasteride.

The effects of HT080 on the expression of Bcl-2 and Bax in BPH-induced rats were investigated, and the results are shown in Figure 7. As shown in the results, the expression of Bcl-2 from the BPH group significantly increased compared to the sham group (*p* < 0.01), while the expression of Bcl-2 from HT080 was significantly reduced in comparison with the BPH group (*p* < 0.01). In addition, the Bax expression in the HT080 group significantly increased in comparison with the BPH group (*p* < 0.05).

### 3.7. Effects of HT080 on Antiandrogenic and Androgenic Activities

We evaluated whether HT080 has antiandrogenic or androgenic properties by using the Hershberger bioassay. Flutamide was used to treat the positive control group. After 10 days of consecutive treatment with flutamide and HT080, the androgen-dependent tissues—CP, SV, LABC, COW, and GP—were weighed, and the results are shown in Table 1. The relative tissue weights of VP, SV, LABC, COW, and GP from the positive control significantly reduced in comparison with the control group (all *p* < 0.001). In rats treated with HT080 (doses of 100, 300, 1000, and 3000 mg/kg), there was no significant difference in the relative weights of VP, SV, LABC, GP, and COW in comparison with the control group. These results suggest that HT080 does not exhibit androgenic and antiandrogenic activities in rats.

## 4. Discussion

We investigated the therapeutic effects of the *C. cassia* and *R. laevigata* mixture (HT080) on BPH for the first time using the TP-induced BPH rat model. In the BPH model, the increase in prostate weight is an important marker during development [42,43]. The increase in prostate weight usually arises from prostate epithelial and stromal hyperplasia. In this study, the increase in prostate weight (1.78-fold) from the BPH group compared with the sham group indicated the success of BPH induction. The prostate weights of the finasteride and HT080 groups were significantly reduced in comparison with the BPH group, demonstrating the ability of HT080 in inhibiting the BPH. It has been reported in animals that *C. verum* bark, which is similar in major components to *C. cassia* bark, and ellagic acid, the major component of *R. laevigata* fruit, are effective in suppressing BPH [31,32]. It is predicted that both *C. cassia* and *R. laevigata* contributed to the inhibitory effects of HT080 on BPH.

TP has been reported to induce rat prostatic hyperplasia, which is manifested by the prostatic epithelial cell layer’s thickness [44]. For the normal rat histology of the prostate, the prostate is clearly visible with a normal epithelial layer, lumens that are filled up with prostatic secretions, and the connective tissue stroma with normal space and normal blood vessels. In the case of the BPH group, TP induced BPH in rats by increasing the growth of epithelial cells and stromal cells, resulting in the increased thickness of the epithelial layer with numerous folds in the prostatic lumen and thereby decreasing the volume of the lumen. After the administration with finasteride and HT080, the histological morphology showed a decrease in epithelial layer thickness without folds in the prostatic lumen in comparison with the BPH control rat prostate. These results suggest that HT080 can inhibit BPH by reducing the growth of prostate epithelial cells.

BPH is a disease related to alterations in the balance of hormones. Testosterone and DHT are the two main androgens related to BPH’s progression. Testosterone is the major androgen in the blood, and DHT is the primary androgen in the prostate gland. These two androgens bind to the AR competitively. This complex of androgen-AR stimulates androgen-regulated processes. As observed in Figure 3, the serum concentrations of testosterone and DHT from the HT080 group were significantly reduced compared to those in the BPH group, suggesting that HT080 has the potential to regulate androgen-AR signaling. In the BPH pathogenesis, DHT is converted from testosterone by 5α-reductase enzymes and then bound to the AR. The AR plays a vital role in the development and maintenance the prostate function; when signaling is blocked, the volume of BPH can decrease and LUTS can be relieved [45,46]. In a previous study, the expression of AR from BPH patients significantly increased compared to the normal prostate, suggesting that AR plays a crucial role in promoting the prostate cell’s proliferation [47]. As seen in Figure 6, the expression of AR was higher in the BPH group compared with the sham group; this result is similar to the previous study [47]. The expression of AR after treatment with HT080 was reduced compared with the BPH group, indicating that HT080 can down-regulate the expression of AR, thereby inhibiting the prostate cell’s proliferation. 

Cell growth is regulated by the balance between cell apoptosis and proliferation. The disruption of the molecular mechanisms in these two processes may lead to BPH [48]. Although the mechanism of BPH is not fully understood, previous studies reported that inducing apoptosis and the inhibition of cell proliferation contributes to the treatment of BPH [49,50]. A decrease in apoptotic activity plays an important role in the BPH pathogenesis [51]. In the apoptosis process, the Bcl-2 family is one of the most characterized protein families; its role in apoptosis has been identified due to its Bcl-2 homology domain and involvement in the regulation of apoptosis [52]. The Bcl-2 family functions include inhibiting apoptosis (anti-apoptotic) and promoting apoptosis (pro-apoptotic). Bcl-2 and Bax are known as two important Bcl-2 family proteins in apoptosis [53]. In normal conditions, the expression of Bcl-2 in prostate tissues is low and related to low apoptosis levels, while the expression of Bax in the prostate is mainly in the secretory epithelial cells and induces apoptosis in response to androgen deprivation [54]. The Bcl-2/Bax ratio determines the apoptotic activity [55]. The imbalance of Bcl-2 and Bax expressions in BPH may affect apoptosis induction. To explore the mechanism associated with apoptosis, Bcl-2 (anti-apoptotic) and Bax (pro-apoptotic) expressions were evaluated after treatment with HT080. Our results (Figure 7) revealed that HT080 decreased anti-apoptotic Bcl-2’s expression and increased pro-apoptotic Bax’s expression compared to the BPH group, thereby decreasing the ratio of Bcl-2/Bax. The results suggest that HT080 has potential in the treatment of BPH by inducing apoptosis.

To confirm the safety of HT080 used in the treatment of BPH, the toxicity of HT080 in the liver was evaluated via ALT and AST, the two most important indices in predicting the risk or toxicity of a drug to the liver. In our study, the safety of HT080 in the treatment of BPH is shown in Figure 5 As a result, there was no significant difference in the values of AST and ALT among the four groups, indicating the normal liver function of rats after HT080 administration [56]. 

Due to BPH being a disease involving imbalanced hormones, the potential androgenic and anti-androgenic activities of treatment drugs need to be determined. Androgens are essential for the maintenance of the reproductive system until death. When a compound acts as an androgen or antiandrogen, it can disrupt the endocrine system and compromise reproductive and developmental processes. The Hershberger bioassay used in this study is a short-term screening assay for evaluating the potential of a compound to exert androgenic or antiandrogenic activities. The effects of HT080 on the androgenic and antiandrogenic activities were evaluated based on the change in relative weights of five androgen-dependent tissues, such as VP, SV, LABC, COW, and GP. As seen in Table 1, significant (*p* < 0.001) decreases in the relative weights of VP, SV, LABC, COW, and GP were observed in the flutamide group compared to the control group. However, there was no significant difference in these variables between the HT080 groups and the control group. These results indicate that HT080 with various doses (100, 300, 1000, and 3000 mg/kg) does not show androgenic and antiandrogenic activities.

## 5. Conclusions

In summary, our results suggest that HT080 has inhibitory effects on BPH, and these beneficial effects might be partially attributed to regulating AR signaling and apoptosis in the prostate. HT080 without androgenic and antiandrogenic activities can be a good candidate for the treatment of BPH.

## Figures and Tables

**Table 1:** Effects of orally administered flutamide and HT080 on androgen-dependent tissue weights of testosterone propionate-supplemented castrated rats.

Androgen-Dependent Tissue Weight/Body Weight (%)					
Treatment	Ventral Prostate	Seminal Vesicles	Glans Penis	LABC	Cowper’s Gland
Control	0.041 ± 0.005	0.121 ± 0.031	0.027 ± 0.004	0.183 ± 0.014	0.010 ± 0.001
Flutamide 3 mg/kg	0.011 ± 0.001 ***	0.017 ± 0.006 ***	0.013 ± 0.003 ***	0.099 ± 0.009 ***	0.004 ± 0.001 ***
HT080 100 mg/kg	0.030 ± 0.009	0.089 ± 0.016	0.026 ± 0.004	0.170 ± 0.011	0.009 ± 0.001
HT080 300 mg/kg	0.035 ± 0.014	0.091 ± 0.038	0.030 ± 0.003	0.165 ± 0.013	0.010 ± 0.003
HT080 1000 mg/kg	0.039 ± 0.038	0.125 ± 0.031	0.031 ± 0.004	0.173 ± 0.034	0.010 ± 0.001
HT080 3000 mg/kg	0.039 ± 0.008	0.127 ± 0.042	0.031 ± 0.003	0.186 ± 0.035	0.010 ± 0.002

*** *p* < 0.001 (compared to control group using Dunnett’s test).

## Data Availability

The data presented in this study are available upon request from the corresponding author.
